# Control of Infectious Diseases in the Era of European Clinical Microbiology Laboratory Consolidation: New Challenges and Opportunities for the Patient and for Public Health Surveillance

**DOI:** 10.3389/fmed.2018.00015

**Published:** 2018-02-02

**Authors:** Olivier Vandenberg, Zisis Kozlakidis, Jacques Schrenzel, Marc Jean Struelens, Judith Breuer

**Affiliations:** ^1^Innovation and Business Development Unit, LHUB-ULB, Pôle Hospitalier Universitaire de Bruxelles, Université Libre de Bruxelles (ULB), Brussels, Belgium; ^2^Centre for Environmental Health and Occupational Health, School of Public Health, Université Libre de Bruxelles (ULB), Brussels, Belgium; ^3^Division of Infection and Immunity, University College London, London, United Kingdom; ^4^The Farr Institute of Health Informatics Research, University College London, London, United Kingdom; ^5^Genomic Research Laboratory, Service of Infectious Diseases, Geneva University Hospitals, Geneva, Switzerland; ^6^Bacteriology Laboratory, Service of Laboratory Medicine, Department of Genetics and Laboratory Medicine, Geneva University Hospitals, Geneva, Switzerland; ^7^Microbiology Coordination Section, Office of the Chief Scientist, European Centre for Disease Prevention and Control (ECDC), Stockholm, Sweden

**Keywords:** clinical microbiology, consolidation of laboratory service, clinical impact, infectious diseases surveillance, public health

## Abstract

Many new innovative diagnostic approaches have been made available during the last 10 years with major impact on patient care and public health surveillance. In parallel, to enhance the cost-effectiveness of the clinical microbiology laboratories (CMLs), European laboratory professionals have streamlined their organization leading to amalgamation of activities and restructuring of their professional relationships with clinicians and public health specialists. Through this consolidation process, an operational model has emerged that combines large centralized clinical laboratories performing most tests on one high-throughput analytical platform connected to several distal laboratories dealing locally with urgent analyses at near point of care. The centralization of diagnostic services over a large geographical region has given rise to the concept of regional-scale “microbiology laboratories network.” Although the volume-driven cost savings associated with such laboratory networks seem self-evident, the consequence(s) for the quality of patient care and infectious disease surveillance and control remain less obvious. In this article, we describe the range of opportunities that the changing landscape of CMLs in Europe can contribute toward improving the quality of patient care but also the early detection and enhanced surveillance of public health threats caused by infectious diseases. The success of this transformation of health services is reliant on the appropriate preparation in terms of staff, skills, and processes that would be inclusive of stakeholders. In addition, rigorous metrics are needed to set out more concrete laboratory service performance objectives and assess the expected benefits to society in terms of saving lives and preventing diseases.

## Background

Infectious diseases currently contribute about 20% of the global annual death causes (1) and 10% of the total disease burden in Europe (2). Within the European Union (EU), public health priorities in this area include antimicrobial resistance (AMR), vaccine preventable diseases, tuberculosis, influenza, and sexually transmitted infections (3, 4). Addressing this challenge, the key role of clinical microbiologists (CMs) in improving appropriate use of antimicrobials in human medicine has been reaffirmed, notably by ensuring timely production and communication of critical diagnostic results and standardized drug susceptibility testing reports in accordance with local treatment guidelines (5). The provision of facility-specific cumulative susceptibility reports for bacterial pathogens against antibiotics on the formulary also forms an essential part of CM work. Additionally, CMs provide daily counseling to clinicians on etiological infection diagnoses and management, including correct sampling for and interpretation of test results, and targeted therapy of difficult-to-treat resistant pathogens and complicated infections. As members of the hospital antimicrobial stewardship team, CMs take on responsibilities that include coordination, planning of infection control activities, post-prescription review, and feedback (6, 7). Hence, CM and clinical microbiology laboratories (CMLs) demonstrate their ability to (i) inform and improve individual patient care, (ii) contribute to outbreak management and hospital infection control, and (iii) provide accurate surveillance data on infectious diseases and AMR. This information can be subsequently used in the review of local treatment guidelines and the design and evaluation of national health policies (8).

In the past decade, clinical microbiology experienced revolutionary advances in terms of culture-independent molecular detection assays, laboratory automation, information systems linkage, and point-of-care testing, to name but a few. The initial optimism associated with these technological strides forward has now entered a phase of realistic discussion on the challenges and complexities posed by clinical translation of microbial genomics (9, 10), bioinformatics (11), economics (12), and other factors (13). These technical developments, together with health-care reforms toward cost-effectiveness call for an overhaul of the operational processes and overall structure of CMLs (14).

At present major emphasis is placed on the operational streamlining and consolidation of CMLs. Through this consolidation, an operational model emerged that combines large centralized CMLs performing most tests on one high-throughput analytical platform connected to several distal laboratories dealing locally with urgent analyses at near point of care (15). The centralization of diagnostic services over a large geographical region created the concept of regional-scale “*microbiology laboratories network*.” Although the volume-driven cost-savings associated with such laboratory networks seem self-evident, the consequence(s) on the quality of patient care and infectious disease surveillance remain less obvious. This article examines from the clinical to the public health perspective the challenges and opportunities of the wide-spread consolidations that the European clinical microbiology sector currently faces.

### Changing the Landscape: Clinical and Financial Perspectives

Laboratory service consolidations have to answer the following: “*How can the consolidation make the laboratory operations more advantageous both financially and clinically, given available resources?*”

From a clinical laboratory perspective, a centralized laboratory network enables addressing different levels of complexity in analytical processes, while concurrently servicing increased volumes of routine tests. The challenge is to simultaneously address the demand for detection and characterization of unusual or exotic pathogens, drug resistance, or virulence traits as appropriate, and meeting the requirement for health-care cost containment and financial efficiency. In this perspective, a number of high profile microbiology services consolidations have already taken place in the private sector over the last decade: (i) BioReference Laboratories, Inc., acquiring Edge BioServ (2013) and merging with OPKO Health (2016); (ii) Sonic Healthcare Limited acquiring CBLPath, Inc. (2010) and an additional ten such acquisitions (2010–2017) in Europe alone; (iii) LabCorp, Inc., with seven such acquisitions (2010–2017); Quest Diagnostics, Inc., with six such acquisitions (2010–2017); and others. It was expected that this trend of CML consolidation would expand to larger academic health-care providers, such as the Karolinska University Hospital merger in Stockholm (2005–2008); the Charité Hospitals mergers in Berlin (2003); or the multi-faceted mergers led by the Royal Free London and University College London Hospitals forming UCL Partners (UCLP, 2005 onward). Due to the projected cost efficiencies and anticipated health-care improvements, regional or local authorities support such consolidation initiatives often in partnership with the academic sector. The laboratory network of the Assistance Publique—Hôpitaux de Paris (AP-HP) or the recent (2015) launch of the University Laboratory of Brussels (LHUB-ULB) are representative examples.

An effective approach includes the creation of task-specific teams representing multiple skills built around infectious diseases specialists and CMs. The need for an increase in professional diversity sometimes contradicts the established specialty training and certification pathways and creates complexities with regard to the required staffing structure and laboratory organization. In general, the utilization of cross-disciplinary teams forced the move in the direction of a flatter hierarchical structure within healthcare organizations (16–18).

Even though CMs play a pivotal role in the management of infected patients (19); the technological advances and laboratory consolidation process have clearly changed the landscape of the interaction between clinicians and CMs. Increasingly, such interactions are based on video conferencing during microbiology rounds and resident teaching (20, 21). However, the effectiveness of such interactions is highly dependent on the ability for a correct understanding and interpretation of the message. Regular telephone calls (anchored by less frequent face-to-face meetings) are still considered effective as they allow for immediacy and a two-way dialog and opinion exchange (22, 23). Clinicians often emphasize the input resulting from the physical presence of CMs on ward rounds as ensuring a strong professional interaction with staff and the understanding of new technological diagnostic options, emerging pathogens or drug resistance mechanisms and local epidemiological trends (24, 25). In terms of education, maintaining the physical presence of CM on satellite clinical sites contributes to the continuing education of staff at those sites. The validation of results from the satellite laboratory owing to a telemicrobiology system is a promising solution making it possible to maintain the physical presence of a microbiologist at distant sites and ensure the satellite lab functions efficiently (26). Nevertheless, telemicrobiology requires efficient communication between teams located in satellite and central labs and a reliable IT department to manage potential connectivity dysfunctions.

### Changing the Analytical Process: From the Cottage Kitchen to the Factory Floor

**Figure 1:**
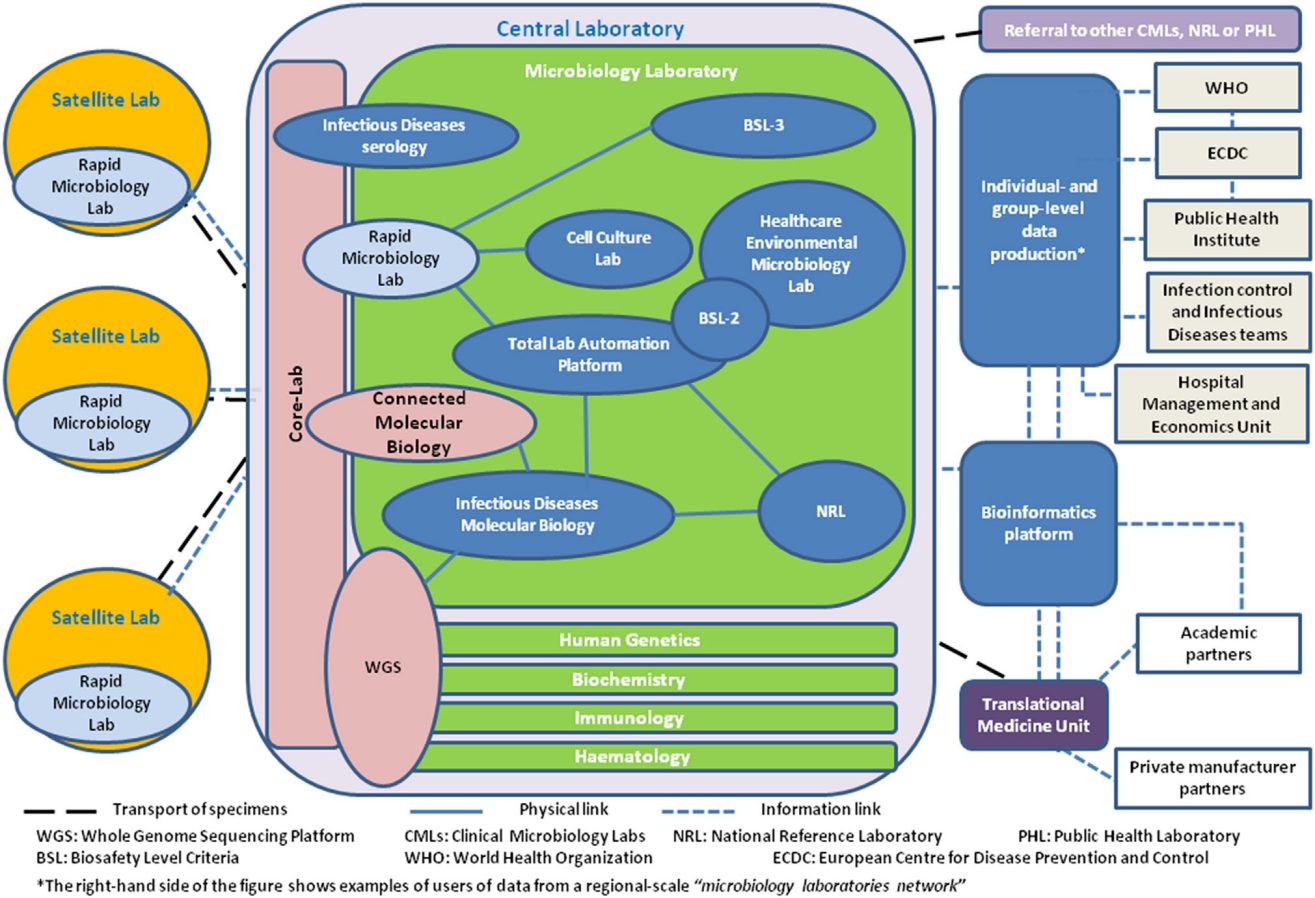
Bubble diagram describing the relationships between the different laboratory, public health, and translational medicine structures in a clinical microbiology network perspective.

According to the European Society of Clinical Microbiology and Infectious Diseases, the number of microbiological services in European countries ranged from 4 to 69 laboratories/10^6^ inhabitants (27). This wide variation supports the argument that further consolidation in CMLs within Europe remains possible. Consolidation frequently results in the adoption of 24/7 working patterns and increased automation, while microbiological services increasingly integrate within wider laboratory services where infectious serology and now molecular testing are often processed by biochemists in a general core-laboratory (28). Consequently, clinical microbiology analysis progressively moves toward an organization relying on different analytical platforms operated independently from the discipline or type of pathogen considered. External linkage to allow collaboration and systematic referral of specimens for further characterization and data to national reference laboratories (NRLs) with a public health mandate is essential. Furthermore, communication and collaboration with food safety, water quality, and environmental laboratories are also playing an important role in a public health microbiology network at national level within the One Health context (29). The relationships between different laboratory structures constituent within a regional clinical microbiology network are summarized in Figure 1.

A major advantage of the consolidated CMLs is the expansion of the range of activities, able to accommodate high technology and sophisticated tests with increased sensitivity and specificity (30), while the usual day coverage is extended through a second (and third) shift. Same-day, direct assays, including molecular assays for selected organisms, are performed as a matter of routine thus reducing time to obtain results (TTR) (31). A potential paradigm shift for core laboratories in the implementation of innovative technologies at high-throughput testing volumes is the widespread deployment of molecular diagnostics in both distal and centralized laboratories. Near-patient testing would include so-called 1- to 2-h “plug-and-play” nucleic acid amplification tests for which a rapid result can directly impact patient care. More-complex/high-volume tests would be dispatched to a core facility. An indicative list of tests that can be performed at peripheral CMLs is shown in Table S1 in Supplementary Material. However, each hospital presenting its own specificity, the stakeholder input in the processing priorities, definition of ideal turnaround times and adequate format of test result reports is central to the success of the consolidation process.

Although the reduction in laboratory testing costs (per-capita or per-analysis) remains a clear motivating force to rationalize operations; CMLs constitute only a small percentage (about 4%) of most hospitals budgets (32). Therefore, cost savings and efficiencies achieved in individual test volumes have a relatively small impact on the overall operating hospital budget. However, as laboratory tests are estimated to impact up to 60 to 70% of medical decisions the major impact of the CMLs consolidation will be indirect through the support of medical decisions both at an individual patient and public health level (33, 34). Such aspects are presented in the following section.

## Clinical Impact

The increasing positioning of health-care providers from condition-centric to patient-centric care impacts the CML organization, workflow, and specimens flow. Microbiology results, once viewed as confirmatory and often delivered after patient management decisions were made, are now integrated into the clinical workflow and decision-making algorithms. In addition, the potential value of the CML itself has been transformed by the superior diagnostic performance of innovative molecular and biophysical technologies (35).

However, improved TTR due to new diagnostic tools, e.g., molecular diagnostics or the Matrix Assisted Laser Desorption Ionization-Time Of Flight Mass Spectrometry, will only have clinical impact if used by without delay; therefore, good communication between health professionals remains fundamental. Only a few studies have evaluated the true impact of rapid microbial identification (RMI) including direct DNA identification from positive blood cultures (36–38). For example, it has been demonstrated that most clinical changes involved treatment escalation in the general patient population and treatment de-escalation in the oncological patient population (39). In the pediatric population, RMI is particularly helpful in quickly confirming contamination by cutaneous bacteria. In some studies, it was shown that the delay in administering the modified treatment was high (>4 h in ap. 50% of cases), suggesting that communication between health professionals can improve further (39). These observations underline the different impact of the RMI across various hospitals, but also emphasize the important role of clinical pharmacists who can contribute to better coordination of care (40).

Furthermore, the recent availability and affordability of high-throughput sequencing methods is expected to impact clinical microbiology. It is now possible to sequence bacterial genomes in less than a week at a cost similar to that of other medical diagnostic procedures. Besides outbreak investigations, genomic applications currently focus on pathogenicity and the pathogen AMR profile (41, 42). There are potential avenues through which such new tools could be incorporated into existing clinical pathways (43). However, the clinical impact of consolidated CMLs needs to consider the broader chain of health-care providers to delineate clinical benefits and costs. In the absence of such broad view, technological CML improvements could equally lead to higher costs without clinical benefits.

## Public Health Impact

As the consolidation of CMLs gathers pace, this change might have a positive impact on the quality of the surveillance information but also alter the ability of the public health authorities to fulfill their missions.

The gradual integration of new molecular detection and typing data into the European surveillance and alert systems represents one of the most exciting and challenging developments that could revolutionize the understanding and enhance control of communicable diseases (44). In order to enable the efficient use of genomic typing technologies, laboratory-based surveillance will need to be linked even more closely with epidemiological data to better detect and monitor outbreaks and improve our understanding of epidemiological changes (45). This deep informational linkage relies upon an extensive system interoperability, where the availability of funds, staff skills, appropriate operational metrics, and management structures are often beyond the reach of individual institutions and require a collective approach. The conversion of health records into electronic form (Electronic Healthcare Records) and the developments of Big Data (General Practitioner records of individual patients’ illnesses and treatments, and data from hospitals about patient attendances, diagnoses, and treatments) at national and EU levels represent new opportunities for facilitating public health efforts (46).

It is conceivable that consolidated CMLs and their regional health-care networks would pave the way to new public health information and cooperation models. Examples of such developments include regional health-care hospital networks with interactive surveillance for AMR control in EU cross-border regions (47). Due to their 24/7 working scheme and advanced automation, consolidated laboratories are also able to provide surge capacity for the analysis of a large influx of samples in the context of an outbreak investigation.

In addition, the ability of networked CMLs to access multiple different partners, geographies, and clinical specialties can enhance their capabilities to provide advanced disease surveillance and early outbreak recognition. For example, in the Brussels region, the LHUB-ULB laboratory structure provided 68% of all infection notifications reported in 2016 to the Belgian sentinel laboratory network (Vandenberg O, unpublished data).

Considering the broad range of scientific expertise and technological capabilities they host, consolidated CMLs may also act as public health reference laboratories. This is the case in Belgium, 26 of the 41 NRL were outsourced through competitive tendering to academic clinical laboratories. A similar figure is found in France where 24 of the 43 NRL are hosted in clinical laboratories (48).

The use of high-throughput whole genome sequencing (WGS) platforms available in large CMLs forms an additional advantage. Outsourced WGS analysis of human pathogens has been recently shown to be portable across countries for supporting multi-state outbreak investigations (49). The availability of sequence-based microbial typing and detection brings about a fundamental change in the sharing of laboratory data, handled and used for medical and public health purposes. Comparative genomic approaches are high-throughput and data-rich and, therefore, create systematic stresses in the collection, analyses, storage, and responsible handling of the generated data. Rather than providing the conventional uni-directional centralized reporting, integration of genomic-derived data to clinical and/or epidemiological databases for infection control and real-time surveillance ultimately requires interactive, secure, information-sharing professional workspaces such as those deployed at national level in the Netherlands with TYPENED (50).

However, the reduction in the number of small clinical laboratories and the aggregation of the remaining ones, may condition the ability to detect epidemiological changes. The sensitivity and representativeness of laboratory-based or confirmed national surveillance systems should be, therefore, carefully assessed using coverage measures, which indicate the proportion of the target population included within the surveillance system. In countries using the “reimbursement system,” the evaluation of the ratio of reimbursed tests performed by the laboratories reporting surveillance data to the total number of tests performed by all laboratories can be used as reliable proxy measure to assess the case ascertainment sampling fraction for these systems (51).

## Impact on Translational Research

The inadvertent increase in volume in clinical microbiological testing has created a substantial and continuous market-driven need for the implementation of high-throughput analytical systems. A number of high profile translational research initiatives (100,000 Genomes Project, UK; the Precision Medicine Initiative, USA; and others globally) are meant to support the eventual technological transfer of high-throughput analytical approaches from research into the CML (52, 53). A characteristic example is the introduction of WGS capabilities into routine health care, which can now support a clinically relevant sample processing turnaround (54) and the high diagnostic granularity needed in complex clinical cases (55). The speed, size, and cost of the equipment has decreased, making the required upfront capital investment by health-care institutions feasible. At the same time, the decrease in the per base cost of sequencing has reduced by 92% from 0.52 to 0.04 USD per DNA Mb (National Human Genome Research Institute, January 2010–January 2015) (56), supporting that operational cost savings can be used to offset the initial capital investment. However, a current bottleneck in the wider adoption of these initiatives is the data analysis and diagnostic interpretation processes which have to be certified, validated, and broadly accepted to be performed routinely and across centers. Consolidated laboratories may help to assess the clinical impact of such new advances by providing the accredited benchmark against which the new methods will be compared (57, 58). A similar approach can be taken for the “lab-on-a-chip” methods (59).

## From Volume to Value: Considerations for Consolidated Clinical Laboratory Models

The impact of CML consolidations has been evaluated so far mainly in the narrow financial sense. It would be particularly interesting to explore the impact of this new model on the wider quality and efficiency of health services, correlating with patient outcome metrics, such as length of hospital stay, mortality, and readmission rates. This broader health system view should include the impact on the clinical management in clinical fields that require significant laboratory input. This suggests the need for more complex studies, not just assessing assay performance for example, but rather entire process performances including the time, expertise and costs both of reporting results and of downstream clinical actions. Beyond the clinical care impact, CMLs also provide key information outputs for the protection of population health, which need to be assessed. In this perspective, the European Centre for Disease Prevention and Control (ECDC) and microbiology experts designed and implemented the EULabCap monitoring system of national public health microbiology performance indicators toward ensuring sufficient capabilities at clinical and reference laboratory levels for well-informed infectious disease prevention and control actions (60).

The availability of increased amounts of high-resolution data at a lower cost creates an anticipation, requirement, and downstream cost(s) for the accommodation, analyses, and interpretation of these data. The inherent systemic flexibility that is necessary to receive different types of data at different speeds and from different locations—and link all that to routinely collected clinical data and report back—is not an insignificant task by itself. A number of questions are raised regarding the new pathways that might be necessary, the different regulatory approaches within Europe to handling this data under the EU personal data protection directives, and data quality issues (46). The ethical implications of big data analysis (BDA) have not been fully explored. In a recent review, Garattini et al. discussed the ethical implication of BDA in terms of loss of individual autonomy and erosion of freedom of choice in response to population-level benefits. If not correctly addressed by the inclusion of ethical design in the creation of big data, such ethical issues might become limiting factors preventing BDA from reaching its full potential (46). The significant role of the CML networks should not be underestimated in the sharing of routine clinical metadata or data collected. Their potential integration into a common data set (biorepositories—as proposed by the Clinical Data Interchange Standards Consortium) (61) would maximize the opportunities for patient contributions to be translated into therapeutic and diagnostic solutions. The consolidation process for example provides a tangible opportunity to extend the scope of pooled analyses of individual patient biomarker data from heterogeneous laboratory platforms and cohorts into population-level studies using merging algorithms (62). At a population level, CML networks can actively support ongoing surveillance, e.g., on AMR, and can add value by connecting some or all of these data (under appropriate management and regulatory structures) to national public health surveillance systems or international networks, such as EARS-Net or the Global Antimicrobial Resistance Surveillance System (GLASS), recently launched by the World Health Organization (63, 64).

Conclusively, the fast changing landscape of CMLs in Europe gives us a range of opportunities to contribute to improving the quality of patient care but also the early detection and enhanced surveillance of public health threats caused by infectious diseases. The success of this transformation of health services is reliant on the appropriate preparation in terms of staff, skills, and processes that would be inclusive of stakeholders. In addition, rigorous metrics are needed to set out more concrete laboratory service performance objectives and assess the expected benefits to society in terms of saving lives and preventing disease.

## Author Contributions

All authors contributed constructively in the conception, drafting, and final approval of the manuscript and agreed to be accountable for all aspects of the work in ensuring that questions related to the accuracy or integrity of any part of the work are appropriately investigated and resolved. The views and opinions expressed herein are the authors’ own and do not necessarily state or reflect those of ECDC. ECDC is not responsible for the data and information collation and analysis and cannot be held liable for conclusions or opinions drawn.

## Conflict of Interest Statement

The authors declare that this manuscript was written in the absence of any commercial or financial relationships that could be construed as a potential conflict of interest. Dr. Vandenberg reports personal fees from BD diagnostics, outside the submitted work. Dr. Schrenzel received grants from Abbott and bioMérieux.
